# Immunological correlates of protection following vaccination with glucan particles containing *Cryptococcus neoformans* chitin deacetylases

**DOI:** 10.1038/s41541-023-00606-0

**Published:** 2023-02-02

**Authors:** Ruiying Wang, Lorena V. N. Oliveira, Diana Lourenco, Christina L. Gomez, Chrono K. Lee, Maureen M. Hester, Zhongming Mou, Gary R. Ostroff, Charles A. Specht, Stuart M. Levitz

**Affiliations:** 1 Department of Medicine, The University of Massachusetts Chan Medical School, Worcester, MA USA; 2 Program in Molecular Medicine, The University of Massachusetts Chan Medical School, Worcester, MA USA

**Keywords:** Protein vaccines, Fungal infection, Preclinical research

## Abstract

Vaccination with glucan particles (GP) containing the *Cryptococcus neoformans* chitin deacetylases Cda1 and Cda2 protect mice against experimental cryptococcosis. Here, immunological correlates of vaccine-mediated protection were explored. Studies comparing knockout and wild-type mice demonstrated CD4^+^ T cells are crucial, while B cells and CD8^+^ T cells are dispensable. Protection was abolished following CD4^+^ T cell depletion during either vaccination or infection but was retained if CD4^+^ T cells were only partially depleted. Vaccination elicited systemic and durable antigen-specific immune responses in peripheral blood mononuclear cells (PBMCs), spleens, and lungs. Following vaccination and fungal challenge, robust T-helper (Th) 1 and Th17 responses were observed in the lungs. Protection was abrogated in mice congenitally deficient in interferon (IFN) γ, IFNγ receptor, interleukin (IL)-1β, IL-6, or IL-23. Thus, CD4^+^ T cells and specific proinflammatory cytokines are required for GP-vaccine-mediated protection. Importantly, retention of protection in the setting of partial CD4^+^ T depletion suggests a pathway for vaccinating at-risk immunocompromised individuals.

## Introduction

Cryptococcosis is an invasive fungal infection caused by fungi of the genus *Cryptococcus*, mainly *C. neoformans* and *C. gattii*. Exposure is thought to most commonly occur following inhalation of aerosolized cells from the environment. In most persons, host defenses are sufficient to kill or contain the fungus in a latent state. However, in susceptible hosts, pneumonia, and life-threatening disseminated infection, especially to the central nervous system can occur^1^. Persons with advanced HIV disease are most vulnerable; however, other immunocompromised individuals with impaired CD4^+^ T cell defenses are also at higher risk, including patients with hematological malignancies, and those on immunosuppressing medications to treat autoimmune conditions, or to prevent rejection following solid organ transplantation^2^. The global burden of HIV-associated cryptococcal meningitis in 2020 was estimated at 152,000 cases, including 112,000 deaths^3^.

Although the incidence and mortality of HIV-associated cryptococcal meningitis have declined in high-income countries, it remains a major health issue in resource-limited areas that have a high prevalence of HIV coupled with insufficient access to diagnostic testing, antiretroviral treatment, and antifungal drugs^2^. Regardless of the setting, once cryptococcal meningitis develops, even with currently available treatments, the morbidity and mortality are substantial^2,4^. Thus, preventative measures, such as the development of efficacious vaccines are urgently needed. However, no licensed anticryptococcal vaccine is available for use^5^.

One approach to cryptococcal vaccine development is the use of whole-organism vaccines that are attenuated by deletion of virulence factors, such as the capsular polysaccharide glucuronoxylomannan, cell wall chitosan, sterylglucosidase, and F-box protein^5–9^. Another approach is engineering *C. neoformans* strains, so they express heterologous murine interferon (IFN)-γ or overexpress zinc finger protein^10–12^. Whole-organism vaccines are relatively easy to manufacture and generally induce good immune responses. Drawbacks, however, include the potential for reactogenicity, autoimmunity, and for live vaccines, infection. Therefore, we have focused on identifying *C. neoformans* protein antigens which can be used in subunit vaccines. Two of the most promising vaccine antigens, Cda1 and Cda2, are members of the chitin deacetylase (Cda) family with no significant homology to human proteins. This, plus their strong immunogenicity and role in catalyzing the deacetylation of chitin to the virulence determinant chitosan make them attractive candidate vaccine antigens^13^. We have demonstrated that glucan particle (GP)-based subunit vaccines, including GP-Cda1 and GP-Cda2 (alone and in combination), could afford a significant survival advantage following pulmonary challenge of mice with the highly virulent *C. neoformans* KN99 strain^14–16^. Some survivors even had undetectable colony-forming units (CFUs) in the lungs at the termination of the experiment.

Here we probed the immunological correlates of GP-vaccine-mediated protection by examining mice with congenital and acquired deficiencies in specific aspects of immune function. We demonstrate CD4^+^ T cells and certain cytokines are crucial for GP-Cda1 and GP-Cda2 vaccine-induced immunity. Furthermore, we investigated the nature of the antigen-specific immune response by ex vivo restimulation of peripheral blood mononuclear cells (PBMC), splenocytes, and lung leukocytes from wild-type (WT) mice that were vaccinated and/or infected. Systemic and durable immune responses were observed in the vaccinated and infected mice, with a robust T-helper (Th) 1 and Th17 response detected in the lung.

## Results

### B cells are dispensable for GP-Cda1 or GP-Cda2 vaccine-mediated protection

**Fig. 1:**
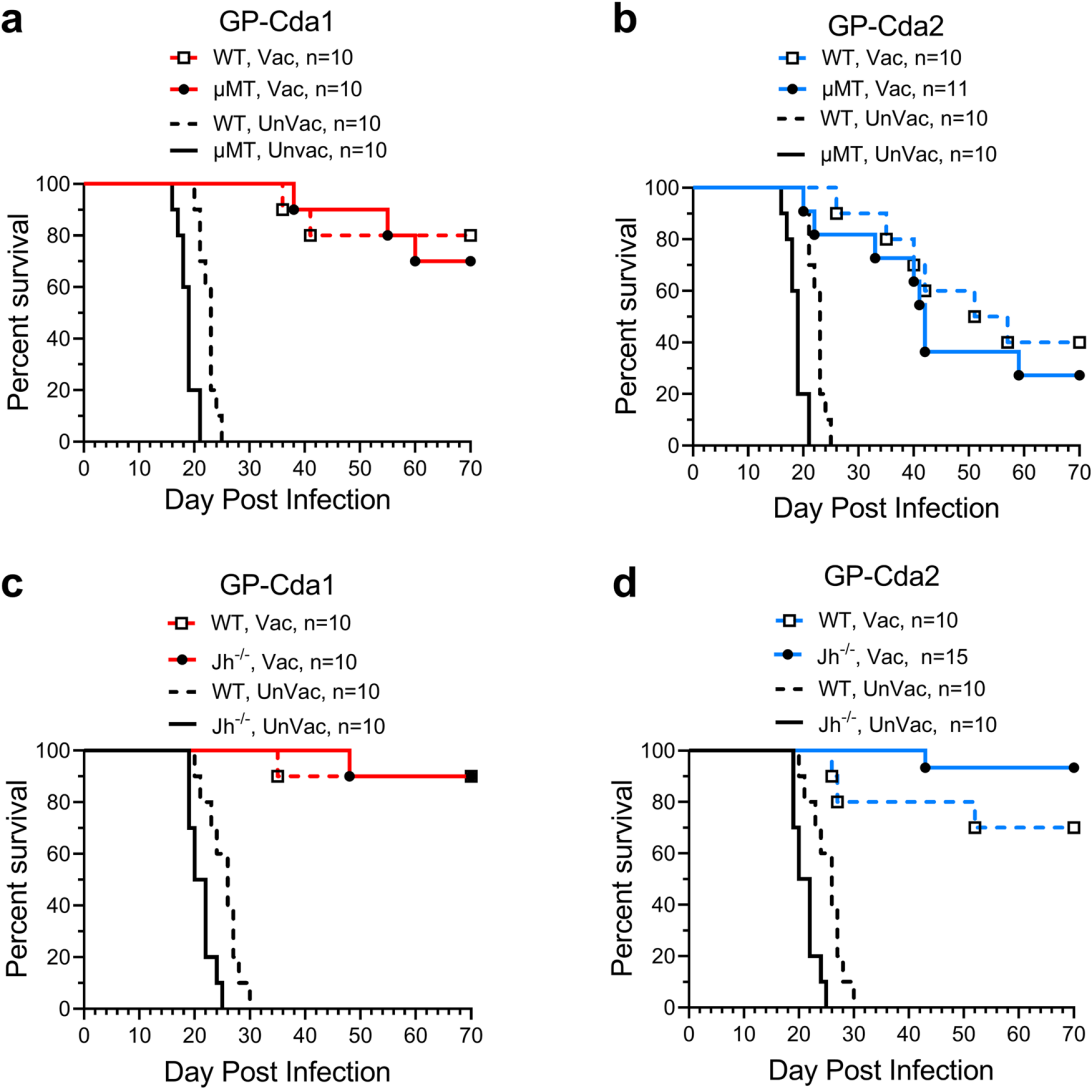
Contribution of B cells to protection by GP-Cda1 or GP-Cda2 vaccines. WT C57BL/6 mice and B cell-deficient (µMT) mice on the C57BL/6 background (**a**, **b**), and WT BALB/c mice and B cell-deficient (Jh^−/−^) mice (**c**, **d**) on the BALB/c background received a prime and two biweekly boosts with the indicated GP-Cda1 or GP-Cda2 vaccine. Two weeks after the last boost, mice were challenged with *C. neoformans* and then followed 70d for survival. Vac vaccinated with GP-Cda1 or GP-Cda2. UnVac unvaccinated. n denotes the number of mice in the experimental group. Note the same survival curves are shown for unvaccinated mice in Fig. 1a, b as well as in Fig. 1c, d. Within each group of WT and mutant mouse strains, *P* < 0.0005 comparing Vac and UnVac mice (**a**–**d**) using Mantel–Cox log-rank test.

In initial experiments, we examined the role of B cells in GP-Cda1 and GP-Cda2 vaccine-mediated protection from pulmonary cryptococcal challenge by comparing two mouse strains congenitally deficient in B cells to their WT counterparts. µMT mice, which have a C57BL/6 background, lack mature B cells and expression of membrane-bound IgM^17^. Following pulmonary challenge with *C. neoformans*, survival of unvaccinated WT and µMT mice was similar, with all mice succumbing to infection by day 25 (Fig. 1a, b). Both GP-Cda1 and GP-Cda2 vaccination significantly protected the WT and µMT mice, with no statistically significant differences noted comparing the mouse strains. Similar results were seen comparing WT BALB/c mice with Jh^−/−^ mice on the BALB/c background. Jh^−/−^ mice carry a deletion of the endogenous murine J segments of the Ig heavy chain locus; as a consequence, they lack mature B cells and have no detectable antibody^18^. Unvaccinated Jh^−/−^ mice succumbed to cryptococcosis by day 25, whereas the WT BALB/c all died by day 30. However, robust protection of Jh^−/−^ mice and WT BALB/c mice was observed following vaccination with either GP-Cda1 or GP-Cda2 (Fig. 1c, d). Thus, vaccine-induced protection is not abrogated by B cell deficiency.

### CD4^+^ but not CD8^+^ T cells are crucial for GP-Cda1 or GP-Cda2 vaccine-induced protection

**Fig. 2:**
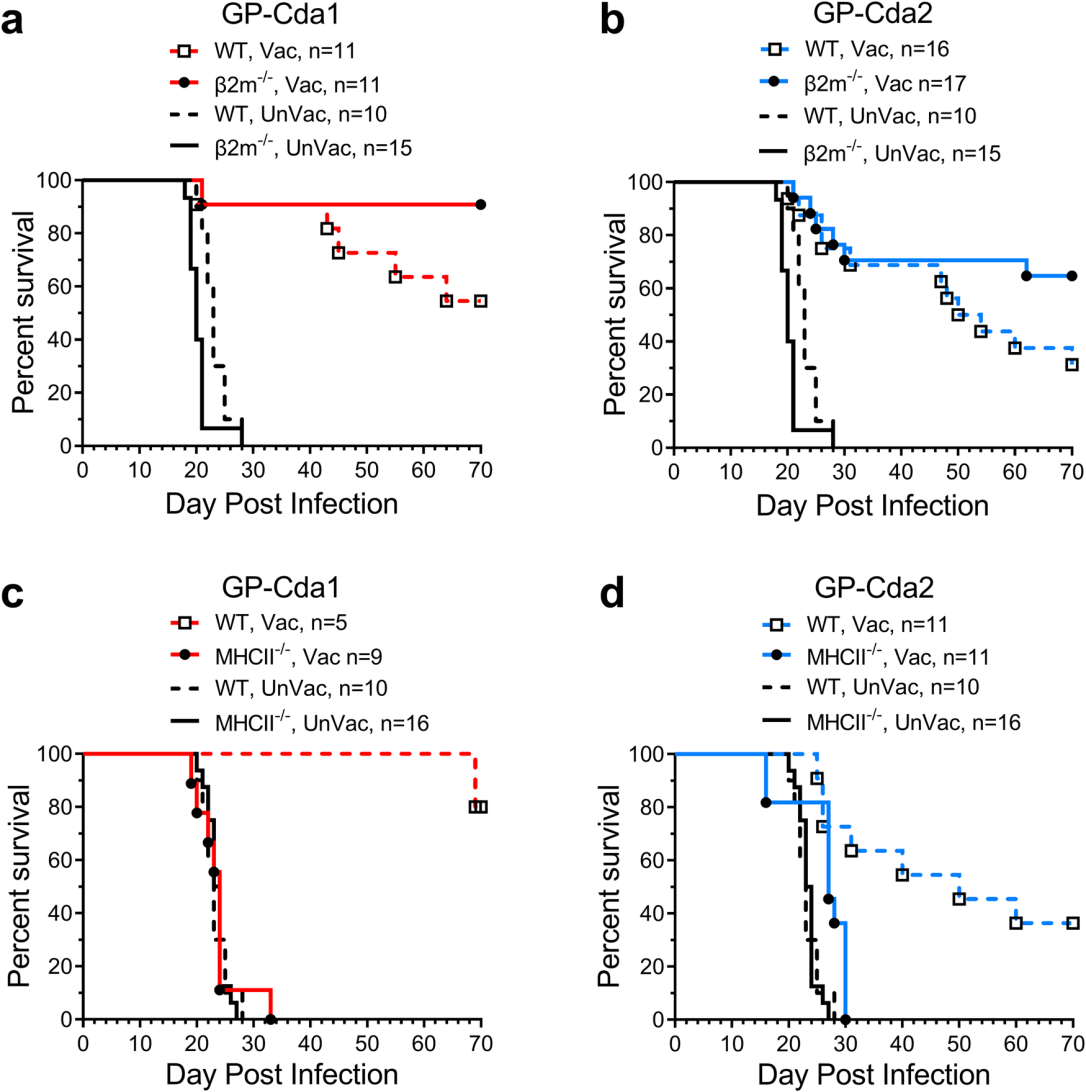
Contribution of CD8^+^ and CD4^+^ T cells to protection by GP-Cda1 or GP-Cda2. Top panel: Wild-type (WT) C57BL/6 mice and CD8^+^ T cell-deficient (β2m) mice on the C57BL/6 background received a prime and two biweekly boosts with GP-Cda1 (**a**) or GP-Cda2 (**b**). Two weeks after the last boost, mice were challenged with *C. neoformans* and followed 70 days for survival. Vac vaccinated with GP-Cda1 or GP-Cda2. UnVac unvaccinated. n denotes the number of mice in the experimental group. Bottom panel: Same as the top panel except for CD4^+^ T cell-deficient (MHCII^−/−^) mice were used (**c**, **d**). Note the same survival curves are shown for unvaccinated mice in Fig. 2a, b as well as in Fig. 2c, d. *P* < 0.005 when comparing WT Vac vs WT UnVac (**a**–**d**), mutant Vac vs mutant UnVac (**a**, **b**, **d**), and WT Vac vs mutant Vac (**c**, **d**) using Mantel–Cox log-rank test.

We next investigated the contribution of T cell subsets to protection mediated by GP-Cda1 and GP-Cda2 vaccines. We first examined whether the CD8^+^ T cell subset contributed to protection in CD8^+^ T cell-deficient β-2-microglobulin knockout (β2m^−/−^) mice. Unvaccinated WT and β2m^−/−^ mice all died 28 days post infection (dpi). However, protection was retained in the β2m^−/−^ mice for both vaccines; if anything, there was a non-significant trend towards enhanced survival in the mutant mice (Fig. 2a, b).

Clinical and experimental studies demonstrate that CD4^+^ T cells are critical for protective immunity against cryptococcosis^19^. Therefore, we next tested our GP-Cda1 and GP-Cda2 vaccines in MHCII^−/−^ mice, which are devoid of all four classical murine MHC Class II chains and consequently lack CD4^+^ T cells. Whether receiving GP-Cda1 or GP-Cda2 vaccination, mice lacking CD4^+^ T cells had 100% mortality by 33 dpi, similar to that seen for unvaccinated mice (Fig. 2c, d). Thus, protection was lost in mice with CD4^+^ T cell deficiency. As expected, protection was retained in the WT-vaccinated mice.

### GP-Cda2 vaccine-mediated protection requires CD4^+^ T cells during both the vaccination and challenge phases of the experiment

**Fig. 3:**
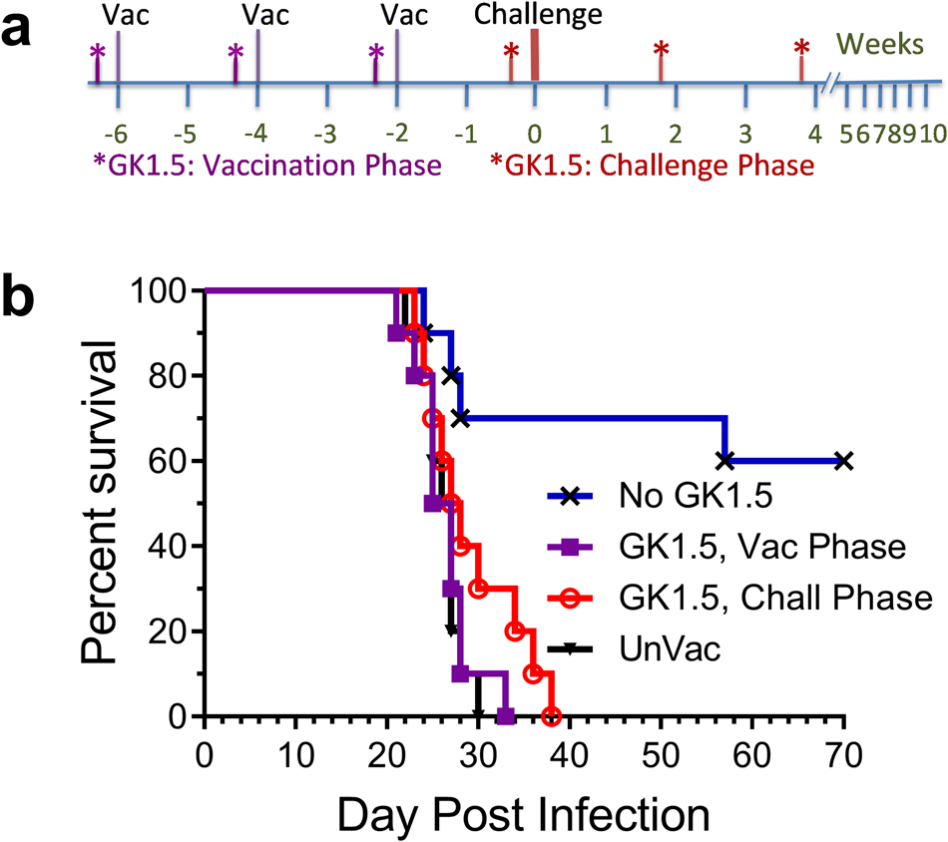
Effect of CD4^+^ T cell depletion during the vaccination and challenge phases on GP-Cda2 protection. **a** Experimental outline. BALB/c mice received a prime and two biweekly boosts of the GP-Cda2 vaccine (Vac) followed by a pulmonary challenge with *C. neoformans*. The CD4-depleting mAb GK1.5 was administered at three biweekly intervals either during the vaccination phase (Vac Phase) 2d before each vaccine dose or the challenge phase (Chall Phase) with the first dose given 2d before *C. neoformans* challenge. Controls included vaccinated mice that did not get GK1.5 (No GK1.5) and unvaccinated (UnVac) mice. **b** Kaplan–Meier survival curve of mice followed for 70 days (10 weeks) after the challenge, with percent survival recorded daily. Data are from two independent experiments, each with 5 mice/group. Significant (*P* < 0.001, Mantel–Cox log-rank test) survival compared with unvaccinated mice was seen only for mice that did not get GK1.5.

**Fig. 4:**
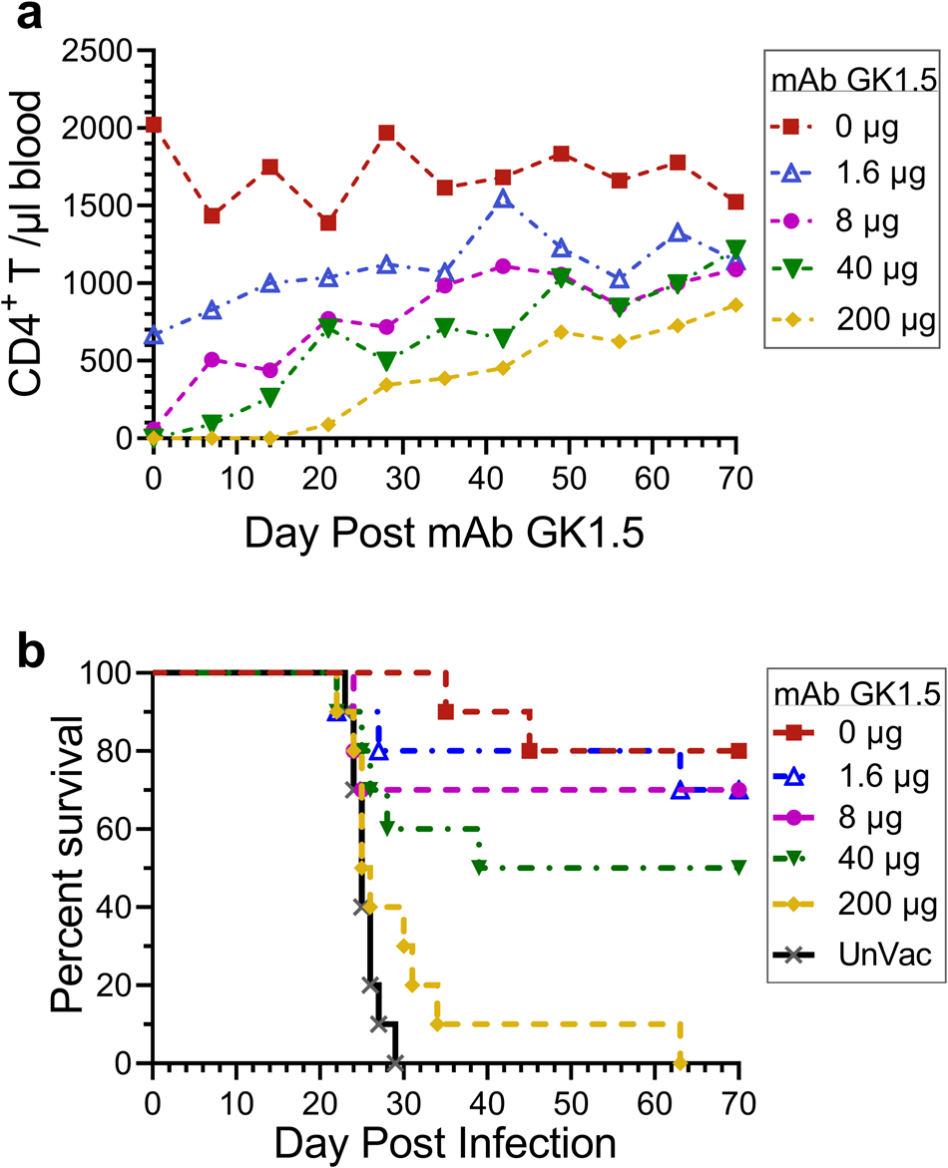
The effect of partial CD4^+^ T cell depletion on vaccine-mediated protection. **a** Naïve BALB/c mice were injected with the indicated amount of anti-CD4 mAb GK1.5 on day −2. Every other week starting on day 0, the mice underwent cheek bleeds to determine the CD4^+^ T cell count in peripheral blood. Four mice per group were studied, staggered so that two mice per group were tested each week. Data were mean values. **b** BALB/c mice were vaccinated thrice at biweekly intervals with GP-Cda1/Cda2. Twelve days after the last boost, mice were injected with the indicated amount of GK1.5. Two days later, the mice received a pulmonary challenge with *C. neoformans* and were monitored for survival until day 70. UnVac unvaccinated. Data were from two independent experiments, each with 5 mice/group. Significant (*P* < 0.005, Mantel–Cox log-rank test) survival compared with unvaccinated mice was seen for those groups of mice receiving a GK1.5 dose ≤40 µg.

As an alternative approach to assess the importance of CD4^+^ T cells for vaccine-induced protection, we studied the effect on survival when the anti-CD4 monoclonal antibody GK1.5 was administered to GP-Cda2-vaccinated mice (Fig. 3). As shown in Fig. 4a, injection of 200 µg of GK1.5 led to nearly complete depletion of blood CD4^+^ T cells for two weeks. Two strategies for CD4^+^ T cell depletion were conducted. In the first, mice were depleted of CD4^+^ T cells in the vaccination phase by giving a dose of GK1.5 2 days prior to each vaccination. For the second strategy, GK1.5 was given two days prior to the fungal challenge and then two additional biweekly injections were given (Fig. 3a). Regardless of whether the GK1.5 was administered during the vaccination or the challenge phase, vaccine-mediated protection was completely lost (Fig. 3b). As expected, the vaccinated mice that did not get GK1.5 survived significantly longer than the unvaccinated mice.

### Retention of GP-Cda1/Cda2 vaccine-mediated protection despite partial CD4^+^ T cell depletion

The data shown in Figs. 2, 3 demonstrate loss of vaccine-mediated protection in the setting of complete CD4^+^ T cell deficiency. However, populations at risk for cryptococcosis generally are only partially deficient in CD4^+^ T cell function. For example, most HIV-positive persons have blood CD4^+^ T cell counts above 100 cells/µL when they are initially found to be HIV-infected or following initiation of antiretroviral therapy^20^. To model whether a vaccine could potentially protect this immunocompromised population, we evaluated doses of anti-CD4 Ab GK1.5 that resulted in an only partial depletion of CD4^+^ T cells. First, using naïve BALB/c mice, we performed a dose-response experiment to determine the kinetics of blood CD4^+^ T cell counts following the administration of GK1.5 (Fig. 4a). Following a single injection of 200 µg GK1.5, CD4^+^ T cells were nearly completely depleted in the blood for two weeks and then gradually increased at subsequent time points. The dose of 40 µg also resulted in profound depletion of CD4^+^ T cells but counts began to recover after one week. Depletion was also seen using the 8 µg and 1.6 µg doses, but the diminution in CD4^+^ T cell counts were more modest. For all doses of GK1.5 tested, CD4^+^ T cell counts never recovered to levels seen in untreated mice.

Next, we immunized mice with the two-antigen combination GP-Cda1/Cda2 vaccine. Two days prior to the cryptococcal challenge, mice were partially depleted of their CD4^+^ T cells using the range of doses tested in Fig. 4a. Mice were then followed over 70 days for survival. Vaccine-mediated protection was inversely proportional to the GK1.5 dose (Fig. 4b). Importantly, all groups of vaccinated mice that received ≤40 µg GK1.5 had ≥50% survival at the end of the study. Taken together with the data in Fig. 3, our results suggest vaccine efficacy is retained in the setting of modest, but not severe, CD4^+^ T cell immunocompromise.

### GP-Cda1/Cda2 vaccination induces systemic and durable antigen-specific immune responses

**Fig. 5:**
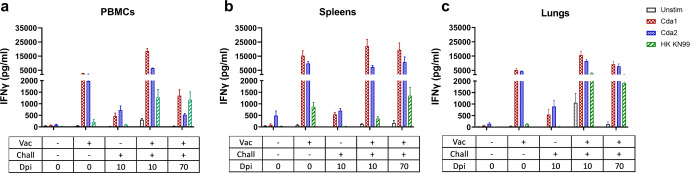
IFNγ production by ex vivo stimulated PBMCs, splenocytes, and lung leukocytes following GP-Cda1/Cda2 vaccination and/or infection. BALB/c mice were vaccinated thrice at biweekly intervals with GP-Cda1/Cda2. Two weeks after the last boost, the mice received a pulmonary challenge with *C. neoformans*. Mice were euthanized at 0 dpi (uninfected), 10 dpi, or 70 dpi. PBMCs (**a**), spleens (**b**), and lungs (**c**) were collected. Controls included unvaccinated mice that were euthanized at 0 dpi or 10 dpi. Single-cell PBMC, spleen, and lung suspensions were prepared following which cells were cultured in complete media supplemented with amphotericin B in the presence of the indicated antigens for either 3 days (PBMC and spleen) or 18 h (lung). Control cells were left unstimulated (Unstim). Supernatants were collected and analyzed for IFNγ by ELISA. Each group had five mice. Data were presented as mean ± SEM. Vac vaccinated with GP-Cda1/Cda2. Chall challenged with *C. neoformans* strain KN99. Dpi days post infection, HK KN99 heat-killed *C. neoformans* strain KN99. IFNγ production following SEB stimulation, as a positive control, was shown in Supplementary Fig. 2. The results of the statistical comparisons between groups were shown in Supplementary Fig. 3.

**Fig. 6:**
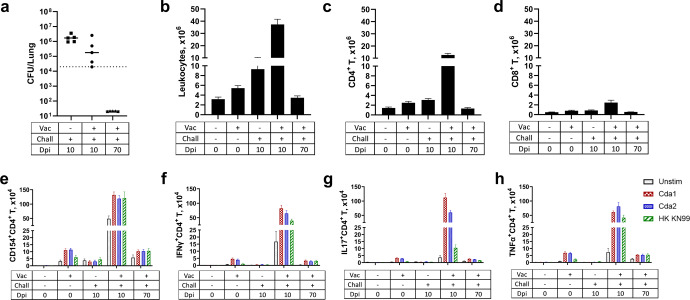
Analysis of lung CFU, leukocytes, and ex vivo antigen-stimulated Th intracellular cytokine production following GP-Cda1/Cda2 vaccination and/or infection. BALB/c mice were vaccinated subcutaneously thrice at biweekly intervals with GP-Cda1/Cda2. Two weeks after the last boost, the mice received a pulmonary challenge with *C. neoformans*. Mice were euthanized at 0 dpi (uninfected), 10 dpi, or 70 dpi. Controls included unvaccinated mice euthanized at 0 dpi or 10 dpi. Lungs were harvested and single-cell suspensions were prepared. **a** CFU/lung were determined. **b** Leukocytes at the interface of a 67 and 40% Percoll gradient were collected and counted. **c**–**h** Leukocytes were cultured in complete media supplemented with amphotericin B and stimulated with indicated antigens or left unstimulated (Unstim) for 18 h. Then the cells were collected, stained, and analyzed by polychromatic FACS, as described in Methods. **c**, **d** The numbers of CD4^+^ T and CD8^+^ T cells were calculated by multiplying the percentage of each population times the total leukocyte count. **e**–**h** The numbers of CD4^+^ T cells expressing the activation marker CD154, or producing the intracellular cytokines IFNγ, IL-17, and TNFα following ex vivo stimulation. Each group had five mice. In Fig. 6a, the bar represents the median of individual lung CFUs, and the dotted line represents the inoculum dosage for a challenge (2 × 10^4^ *Cryptococcus*). In Fig. 6b–h, data are presented as mean ± SEM. Vac vaccinated with GP-Cda1/Cda2, Chall challenge with *C. neoformans*, Dpi days post infection, HK heat killed. Statistical comparisons between groups are shown in Supplementary Fig. 5.

For the next two sets of experiments (Figs. 5, 6), we sought to determine the nature of the adaptive immune response to vaccination and infection. To do so, ex vivo antigen-stimulated responses were investigated in five groups of BALB/c mice: (1) unvaccinated, unchallenged; (2) vaccinated, unchallenged; (3) unvaccinated, challenged, euthanized 10 dpi; (4) vaccinated, challenged, euthanized 10 dpi; (5) vaccinated, challenged, and euthanized 70 dpi. The vaccine administered was the GP-Cda1/Cda2 combination. The ex vivo antigenic stimuli consisted of purified Cda1 or Cda2 protein produced in *E. coli*, and heat-killed *C. neoformans*. Control cells were left unstimulated.

The first set of experiments measured IFNγ secretion by antigen-stimulated PBMCs (Fig. 5a), splenocytes (Fig. 5b), and lung leukocytes (Fig. 5c) harvested from the five groups of mice. This cytokine was chosen as it is thought to be crucial for the clearance and control of the mouse and human cryptococcal infection^21,22^. When comparing cells from the three anatomical sites, similar qualitative patterns of IFNγ release were seen. As expected, antigen-stimulated cells from the naïve mice had low to undetectable levels of IFNγ production. Cells from vaccinated but uninfected mice, obtained two weeks following the third vaccination, had remarkable IFNγ secretion following ex vivo stimulation with the vaccine antigens, Cda1 and Cda2. In contrast, IFNγ release following stimulation with heat-killed (HK) *C. neoformans* was much lower. When considering cells from mice that were infected but not vaccinated, Cda1 and Cda2 stimulated modest amounts of IFNγ that were considerably lower than what were observed in the vaccinated group. For cells from mice that had been vaccinated and then harvested at 10 dpi, the recall immune response to Cda1 and Cda2 was similar to that seen in mice that had been only vaccinated. However, when stimulated with HK *C. neoformans*, PBMCs and lung cells had more robust IFNγ release compared with the corresponding cells from the vaccination-only group. Finally, of the five mice in the vaccinated group followed for 70 days after infection, all were alive with undetectable lung CFUs (Fig. 6a) at the termination of the study. Nevertheless, recall immune responses persisted in cells from all three anatomical sites, albeit at lower levels in the PBMCs. Overall, these data indicate that GP-Cda1/Cda2 vaccine-induced immunity is systemic and long-lasting.

### GP-Cda1/Cda2 vaccinated mice have robust antigen-specific Th1 and Th17 responses in the pulmonary compartment following the cryptococcal challenge

As the lungs are sites of initial infection after natural exposure to airborne *Cryptococcus* by inhalation, the immune response in the pulmonary compartment is crucial for vaccine-induced protection. Thus, we set up the second set of experiments and further analyzed the lungs from the same groups of mice described in Fig. 5. Fungal clearance, numbers of leukocytes, CD4^+^ T cells and CD8^+^ T cells, T cell activation, and ex vivo antigen-stimulated intracellular cytokine production were studied.

At 10 dpi, although not statistically significant, there was a trend toward better fungal control in the lungs of vaccinated compared to unvaccinated mice (Fig. 6a). Notably, mice that survived to the end of the study had undetectable lung CFUs. In mice vaccinated and infected, a remarkable, tenfold influx of leukocytes, including CD4^+^ T cells, into the lung was observed at 10 dpi (Fig. 6b, c). At 70 dpi, the numbers of leukocytes and CD4^+^ T cells were similar to values seen in uninfected mice. CD8^+^ T cell numbers in the lung also increased in vaccinated mice at 10 dpi compared with naïve or unvaccinated infected mice; however, the extent of the increase was considerably more modest compared to that seen with CD4^+^ T cells (Fig. 6d).

We next examined the profile of the CD4^+^ T cell response in the lungs following subcutaneous vaccination and pulmonary infection (Fig. 6e–h). The lungs of mice that were vaccinated but not infected had a non-significant trend towards increased numbers of antigen-stimulated CD4^+^ T cells expressing the activation marker CD154, and the intracellular cytokines IFNγ, interleukin (IL) -17, and tumor necrosis factor (TNF) α in comparison to unstimulated cells. In the lungs of mice that were both vaccinated and infected, at 10 dpi there were dramatic increases in numbers of CD4^+^ T cells expressing CD154, IFNγ, IL-17, or TNFα after antigen stimulation, regardless of whether the antigen was Cda1, Cda2, or HK KN99. These numbers decreased at 70 dpi to levels that were similar to those observed in vaccinated but uninfected mice. Lung CD8^+^ T cells producing IFNγ following ex vivo antigen stimulation were also found in vaccinated mice 10d post infection, albeit in considerably lower numbers compared to what was observed with CD4^+^ T cells (Supplementary Fig. 4a). However, we did not detect significant numbers of CD8^+^ T cells producing IL-17 or TNFα after stimulation (Supplementary Fig. 4b, c).

### Contribution of specific cytokines to protection mediated by GP-Cda1 and GP-Cda2 vaccination

**Fig. 7:**
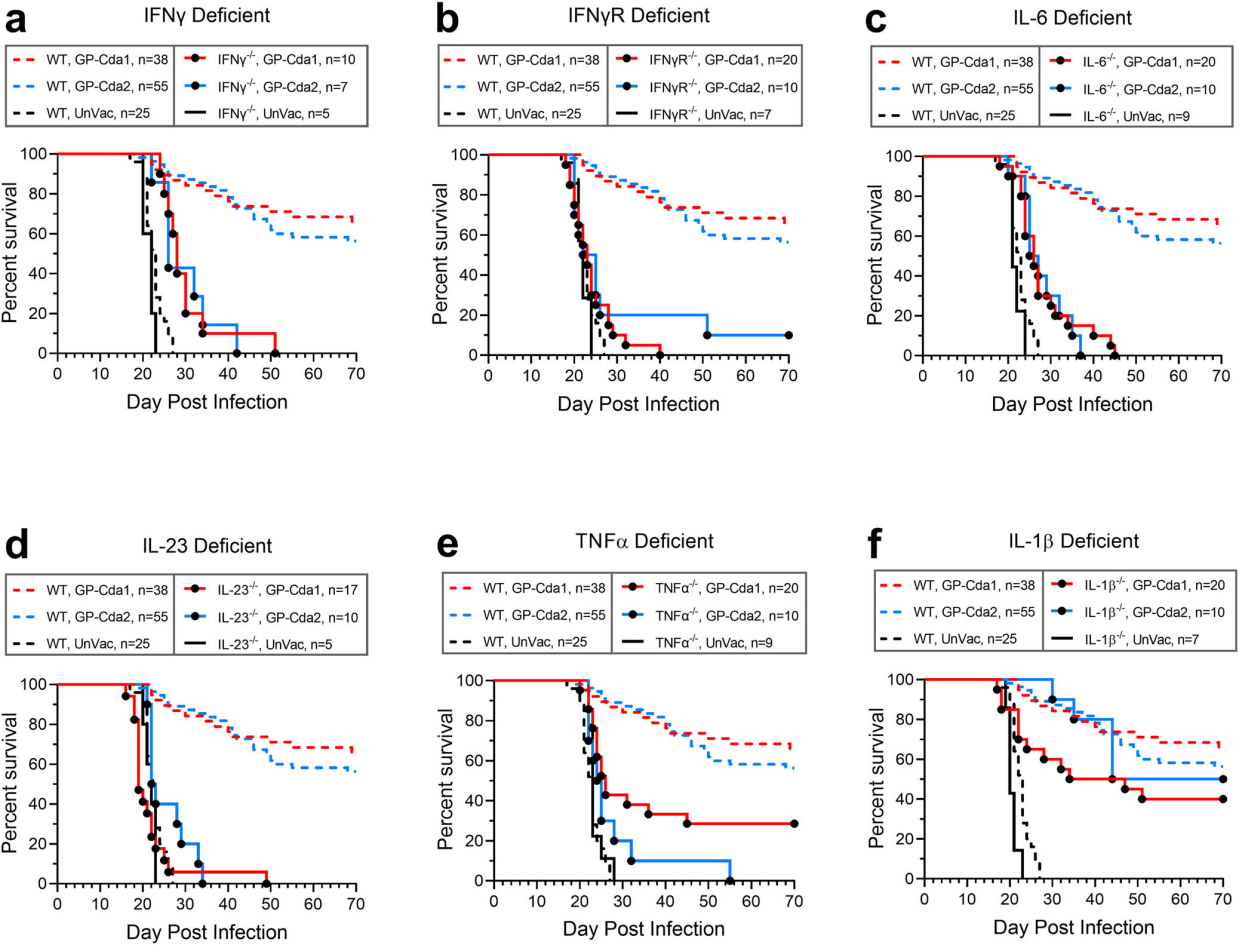
Effect of host gene deletions in selected cytokines or cytokine receptors on GP-Cda1 or Cda2 vaccine efficacy. Wild-type (WT) mice and mice with selected cytokine or cytokine receptor deficiency (**a**. IFNγ^−/−^; **b**. IFNγR^−/−^; **c**. IL-6^−/−^; **d**. IL-23^−/−^; **e**. TNFα^−/−^; **f**. IL-1β^−/−^) received a prime and two biweekly boosts with GP-Cda1 or GP-Cda2. Two weeks after the last boost, mice were challenged with *C. neoformans* and followed for 70 days for survival. UnVac unvaccinated. n denotes the number of mice in the experimental group. All mice were on the C57BL/6 background. Note the same survival curves are shown for WT mice in Fig. 7a–f. For Fig. 7a–e, *P* < 0.001 (Mantel–Cox log-rank test) comparing survival between knockout and WT mice vaccinated with either GP-Cda1 or GP-Cda2. For Fig. 7f, *P* < 0.05 (Mantel–Cox log-rank test) comparing GP-Cda1-vaccinated IL-1β^−/−^ and WT mice.

The intracellular cytokine data demonstrate the capacity of pulmonary T cells from vaccinated and infected mice to produce specific cytokines in response to antigenic stimulation but do not prove that these cytokines are essential to vaccine-mediated protection. Thus, in the final set of experiments, we explored GP-Cda1 and GP-Cda2 vaccine-induced protection in a panel of mice with genetic deficiencies in selected cytokines and a cytokine receptor implicated in host defenses against cryptococcosis. As the knockout mice were on the C57BL/6 background, WT C57BL/6 mice were used as controls. Consistent with our published data^16^, WT C57BL/6 mice vaccinated with GP-Cda1 and GP-Cda2 were significantly protected from *C. neoformans* challenge (Fig. 7). Protection was completely or nearly completely lost in mice deficient in IFNγ (Fig. 7a), IFNγ receptor (IFNγR; Fig. 7b), IL-6 (Fig. 7c) and IL-23 (Fig. 7d), regardless of whether the mice received the GP-Cda1 or GP-Cda2 vaccine. For vaccinated mice deficient in TNFα (Fig. 7e), the picture was more complicated. GP-Cda1 vaccination protected 30% of the TNFα^−/−^ mice but GP-Cda2 vaccination did not elicit protection. Finally, IL-1β^−/−^ mice (Fig. 7f) were significantly protected by both vaccines, although the GP-Cda1 vaccine protected the WT mice significantly better than the mutant mice.

## Discussion

The mechanisms by which a vaccine elicits protective responses against its target pathogen are important to decipher as this knowledge may help predict which populations are likely to benefit. This is especially true for vaccines designed to protect persons with immunocompromise; for such populations, an ideal vaccine would stimulate responses in the parts of the immune system that are relatively intact. For cryptococcosis, in the absence of vaccination, the development of an adaptive CD4^+^ T cell response is critical for host defenses against natural and experimental infections^23,24^. However, other arms of the immune system, including B cells and CD8^+^ T cells have been shown to play supportive roles^25,26^. In the present study, we systematically examined the immunological mechanisms by which GP-based vaccines containing Cda1 and Cda2 protect against experimental cryptococcosis in mice.

Evidence for the importance of antibody responses in protection against cryptococcosis includes mouse and human studies associating antibody responses and Fc receptor polymorphisms with the risk of developing cryptococcal infections^27,28^. In addition, passive administration of monoclonal antibodies directed against the cryptococcal capsular polysaccharide, glucuronoxylomannan (GXM), or vaccination with GXM conjugated to a carrier protein, prolonged survival or reduced fungal burdens in some mouse models of cryptococcosis^29–31^. However, in our studies, protection against cryptococcosis mediated by the GP-Cda1 and GP-Cda2 vaccines was retained in two different B cell deficiency mouse stains: µMT mice on the C57BL/6 background and Jh^−/−^ mice on the BALB/c background. These data indicate that antibody responses are not essential for anticryptococcal immunity elicited by the two GP-vaccines studied here. Similarly, Aguirre et al. found that µMT mice and WT C57BL/6 mice had comparable susceptibility to cryptococcosis regardless of whether the mice had received a sublethal intratracheal immunization with a live *C. neoformans* strain^32^. In another study, protection mediated by an IFNγ-producing *C. neoformans* vaccine strain was retained in B cell-deficient CD19^−/−^ mice^33^. Thus, B cells appear to be dispensable for multiple cryptococcal vaccine candidates under development.

Similar to B cells, our data demonstrate that CD8^+^ T cells are dispensable for GP-Cda1 and GP-Cda2 vaccine-mediated protection. Vaccinated β2m^−/−^ mice, deficient in CD8^+^ T cells, survived as well as the vaccinated WT mice following lethal challenge with *C. neoformans*. In fact, there was a trend towards increased protection in the CD8-deficient mice. The reasons for this trend are speculative but could be related to the compensatory effects of other immune cell components. In addition, although IFNγ-producing CD8^+^ T cells (Tc1) were found in GP-Cda1/Cda2 vaccinated and infected mice lungs, their numbers were considerably lower compared with the numbers of infiltrating CD4^+^ T cells. In contrast, studies using primary pulmonary infection models found that although not playing a dominant role, CD8^+^ T cells contributed to protective immunity and cryptococcal clearance by mediating cellular recruitment, synergizing with CD4^+^ T cells, secreting inflammatory cytokines, and lysing *Cryptococcus*-laden phagocytes^34–36^.

While B cells and CD8^+^ T cells were not required, two lines of evidence demonstrate the non-redundant contribution that CD4^+^ T cells make to GP-vaccine-mediated protection. First, vaccination with GP-Cda1 and GP-Cda2 failed to protect CD4^+^ T cell-deficient MHCII^−/−^ mice against cryptococcal challenge. Second, vaccine-mediated protection was lost when mice were depleted of CD4^+^ T cells using a monoclonal antibody targeting CD4. Notably, protection was abrogated regardless of whether the CD4^+^ T cells were depleted at the time of vaccination or during infection. These results are in marked contrast to what was observed with an attenuated *Blastomyces dermatitidis* vaccine which protected CD4^+^ T cell-deficient mice against blastomycosis and histoplasmosis by eliciting IL-17-producing CD8^+^ T cells (Tc17)^37^. However, no compensatory effect of CD8^+^ T cells was observed in GP-Cda1 or GP-Cda2 vaccinated CD4-deficient mice in our study, reflecting the non-essential role of CD8^+^ T cells in this model.

As CD4^+^ T cells are required for GP-vaccine-mediated immunity, these findings raise the question of whether the immunocompromised populations most at risk for cryptococcosis could still benefit from the vaccine. Taking into consideration that most immunocompromised individuals are only partially deficient in CD4^+^ T cell number or function^20,38^, we established a partial CD4 deletion animal model using a range of doses of the anti-CD4 mAb, GK1.5. The protection elicited by the GP-Cda1/Cda2 combination vaccine was inversely correlated with GK1.5 dosage. Importantly, mice with very low levels of blood CD4^+^ T cells at the time of challenge were protected. The translational significance of these findings remains speculative. It may be possible to vaccinate persons living with HIV while their CD4^+^ T cells counts are relatively high, either at early diagnosis or after antiretroviral treatment. In a recent multicenter trial of patients with cryptococcal meningitis conducted in sub-Saharan Africa, 64% of trial participants had previously received antiretroviral therapy^39^. Other potential *Cryptococcus* vaccine recipients include patients for whom immunosuppression is anticipated in the future, such as individuals on solid organ transplant waiting lists. Finally, it is also worth noting that many cases of cryptococcosis are thought to be due to reactivation of latent disease^40,41^. In these individuals, vaccination could stimulate immune responses to eliminate latent cryptococcal foci.

The durability and pattern of compartmentalization of vaccine-induced immunity are dependent, at least in part, on the vaccine antigens, delivery system, and route of administration^5,42,43^. To explore how adaptive immunity develops after GP-subunit vaccination and pulmonary cryptococcal infection, IFNγ secretion was measured following ex vivo antigen stimulation of immune cells from blood, spleen, and lung leukocytes. Robust antigen-specific immune responses were detected in all compartments for vaccinated mice with or without infection. Of note, the 70d survivors of vaccination and infection still had strong responses, thus suggesting that GP-Cda vaccines elicit durable local and systemic immunity in the experimental pulmonary infection models.

The CD4^+^ T lymphocyte subsets Th1 and (to a lesser extent) Th17 have been associated with protective responses to cryptococcal infection. Postulated mechanisms include activation and recruitment of classically activated (M1) macrophages, dendritic cell maturation, and stimulation of proinflammatory cytokine and chemokine production^44–46^. We have demonstrated that GP-based vaccines elicit strong antigen-specific Th1- and Th17-biased responses in mice and rats^47–49^. Consistent with these observations, following GP-Cda1/Cda2 vaccination and infection, mouse lungs had a robust influx of activated CD4^+^ T cells that produced IFNγ (Th1), IL-17 (Th17), and TNFα following ex vivo antigen stimulation. Moreover, significantly enhanced IFNγ concentrations in ex vivo culture supernatants were detected by ELISA in mice both vaccinated and challenged. These results indicate CD4^+^ T cells are an important source of IFNγ, although the modest increase of Tc1 cells suggests a supplementary role for CD8^+^ T cells. Interestingly, for mice vaccinated but not infected, increased levels of IFNγ were also observed following antigen stimulation. However, the numbers of Th1 and Tc1 cells were not elevated to the degree seen with vaccinated and infected mice, thus suggesting the contribution of other cellular sources of IFNγ, such as group 1 innate lymphoid cells (ILC1), natural killer (NK) cells, B cells, macrophages, and dendritic cells^50^.

Regardless of the cellular source, IFNγ was required for GP-Cda1- and GP-Cda2-induced protection from cryptococcal challenge, as evidenced by the near total loss of protection in mice genetically deficient in this cytokine or its receptor, IFNγR. The binding of IFNγ to IFNγR on macrophages activates a signal inducer and activation of transcription 1 (STAT1) dependent signaling pathway leading to transcription of IFN-stimulated genes^51^. The resulting activated and M1-polarized macrophages produce increased reactive oxygen and nitrogen species with activity against *C. neoformans*^52^. However, IFNγR is expressed on many cell types other than macrophages, and IFNγ promotes DC maturation and drives Th1 responses^51^. Therefore, the role of IFNγ or IFNγR in vaccine-mediated protection is likely multifactorial.

Survival curves similar to those seen in unvaccinated mice were also observed in mice null for IL-6 and IL-23. These two cytokines have a myriad of immune functions including promoting Th17 development and are required for optimal host defenses against cryptococcosis^53–56^. For the other two cytokine knockout mice we studied, TNFα^−/−^ and IL-1β^−/−^, more complex phenotypes were seen with results depending upon the vaccine tested. The GP-Cda1 vaccine partially protected TNFα^−/−^ and IL-1β^−/−^ mice, with survival intermediate between that seen in unvaccinated and vaccinated WT mice. For the GP-Cda2 vaccine, protection was largely lost in the TNFα^−/−^ mice, but similar to WT survival curves in the IL-1β^−/−^ mice. The differential protection observed in the knockout mice when comparing the two vaccines raises the possibility that the vaccines activate different intracellular pathways. TNFα is produced following phagocytosis of *C. neoformans* and is thought to have a central role in host defenses against cryptococcosis^21,57^. IL-1β is released by mononuclear phagocytes, including dendritic cells, following activation of both the canonical NLRP3–ASC–caspase-1 inflammasome and the non-canonical NLRP3–ASC-caspase-8 inflammasome^58^.

In summary, our preclinical studies illuminate the arms of the immune system which are required for GP-Cda1 and GP-Cda2 vaccines to protect mice from cryptococcosis. Using models of congenital and acquired deficiency, a crucial, non-redundant role was found for CD4^+^ T cells. Moreover, vaccinated and infected mice had a robust pulmonary influx of CD4^+^ T cells expressing IFNγ, IL-17, and TNFα. Mice deficient in these cytokines or in pathways leading to their production or responsiveness were no longer protected by vaccination. A major challenge will be bringing vaccines to the human populations most in need. Our discovery that mice are still protected in the setting of partial CD4^+^ T cell depletion provides encouragement that GP-vaccines could still be employed in some immunodeficient hosts. However, to maximize protection in hosts with CD4^+^ T cell impairment, it may be necessary to stimulate other arms of the immune system. One such strategy which we are exploring is studying whether vaccines containing cryptococcal protein antigens which are exposed on the capsular surface will stimulate protective opsonophagocytic antibody responses.

## Methods

### Chemicals and reagents

Chemical reagents were purchased from Thermo Fisher (Pittsburgh, PA), unless otherwise specified. Media for culturing *Cryptococcus* were either Sabouraud dextrose agar, or yeast extract-peptone-dextrose (YPD), containing Difco yeast extract, Bacto peptone, and dextrose with and without 2% agar. Bovine serum albumin was added to 1X phosphate buffered saline (PBS) at a concentration of 0.5% as FACS buffer for flow cytometry staining. The complete medium for mouse cell culture was RPMI 1640 supplemented with 10% fetal bovine serum (FBS), 1% HEPES, 1% GlutaMAX, and 1% Penicillin–Streptomycin. Where indicated, 0.5 µg/ml amphotericin B was included in the complete medium.

### Mouse strains

Experiments were performed using 6–10 weeks old male and female mice in approximately equal numbers. BALB/c and C57BL/6 WT mice were obtained from The Jackson Laboratory (Bar Harbor, ME). B cell-deficient Jh^−/−^ mice on the BALB/c background were purchased from Taconic Biosciences (Rensselaer, NY). B cell-deficient (µMT, JAX stock #002288), CD4-deficient (MHCII^−/−^, JAX stock #003239), and CD8-deficient (β2m^−/−^, JAX stock #002087) mice on the C57BL/6 background were obtained from The Jackson Laboratory. Knockout mice on the C57BL/6 background deficient in IFNγ (IFNγ^−/−^, JAX stock #002287), IFNγR (IFNγR^−/−^, JAX stock #003288), TNFα (TNFα^−/−^, JAX stock #005540), IL-1β (IL-1β^−/−^, JAX stock #068082), IL-6 (IL-6^−/−^, JAX stock #002650), and IL-23p19 (IL-23^−/−^) were also obtained from The Jackson Laboratory, except for the IL-23^–/−^ mice which were from Nico Ghilardi (Genentech, South San Francisco, CA)^59^. Mice were bred and housed in specific pathogen-free conditions in the animal facilities at the University of Massachusetts Chan Medical School (UMCMS). All animal care and procedures were in accordance with protocols approved by the UMCMS Institutional Animal Care and Use Committee.

### *Cryptococcus neoformans*

*C. neoformans var. grubii* strain KN99α^60^, hereafter referred to as KN99, was preserved in 50% glycerol at −80 °C. To prepare fungal cells for infection, KN99 was grown on YPD agar for 48 h at 30 °C, followed by overnight culture in 4 mL liquid YPD at 30 °C with shaking. Cells were washed three times with 1X PBS buffer, and the concentration of yeast cells was determined using a T20 automated cell counter (Bio-Rad, Hercules, CA). CFUs were quantified by culturing on Sabouraud dextrose agar.

To prepare heat-killed (HK) *C. neoformans* as stimuli for ex vivo experiments, KN99 was shaken for 18 h in YPD liquid medium at 30 °C, then diluted 1:200 into fresh YPD medium and shaken for 48 h. The number of cells was determined and the culture was diluted with PBS to 2.6 × 10^7^ cells/ml which is equivalent in dry weight to 1 mg/ml. The fungi were heat-killed at 70 °C for 30 min, and then aliquoted without further washing and stored at −80 °C until use. The complete fungal killing was confirmed by the absence of CFU following plating on Sabouraud dextrose agar.

### GP-vaccines

Cryptococcal proteins Cda1 and Cda2 were expressed in *E. coli* and purified as described in ref. ^16^. The cDNAs encoding for Cda1 and Cda2 protein were synthesized and cloned in the pET-19b vector by GenScript (Piscataway, NJ). Recombinant proteins were purified on HisBind resin (MilliporeSigma, Burlington, MA) and dialyzed against 6 M urea/20 mM Tris-HCl, pH 7.9. Protein concentrations were determined using the bicinchoninic acid (BCA) assay. Protein purity was assessed by SDS-PAGE (Bio-Rad) followed by staining with InstantBlue Coomassie Protein Stain (Abcam, Cambridge, UK). Proteins were concentrated using Amicon Ultra-15 centrifugal filters (10 K MWCO; MilliporeSigma) and adjusted to a concentration of 10 mg/ml. Encapsulated GP-antigen vaccines were prepared by absorbing 5 μl of concentrated Cda1 or Cda2 antigen/ mg of dry GP, lyophilizing, and adding 5 μl of mouse serum albumin (MSA; 50 mg/ml, Equitech-Bio, Kerrville, TX) /mg GP-antigen, lyophilizing, and forming an antigen-MSA-yeast RNA (yRNA) complex to trap the antigen by swelling the GP-antigen-MSA particles in 25 mg/ml yRNA (MilliporeSigma), as described^47^. After washing in saline, each vaccine dose consisted of a 100 μl sterile 0.9% saline suspension of 200 µg GPs (approximately 10^8^ GP particles) containing 10 µg of recombinant protein and 25 µg of MSA complexed with yRNA. Vaccines were characterized for antigen identity and percent antigen encapsulation (>95%) by SDS-PAGE and particle number by hemacytometer.

### Immunization and infection strategy

Vaccines were administered to mice subcutaneously near the midline of the abdomen as a prime dose followed by two boosters, with a 2-week interval between each injection. The GP-Cda1/Cda2 two-antigen combination vaccine followed the same schedule and was formulated such that a single 100 μl dose consisted of a 1:1 mix of GP-Cda1 and GP-Cda2 containing 5 µg of each protein. Two weeks after the last vaccination, mice were anesthetized with 2% isoflurane (Covetrus, Portland, ME) inhalation and orotracheally inoculated with *C. neoformans* suspended in 50 µl PBS. The inoculum was 2 × 10^4^ yeast cells for WT and knockout mice on the BALB/c background, and 1 × 10^4^ cells for WT and knockout mice on the C57BL/6 background. For the survival studies, mice were monitored daily until 70 dpi at which point the experiment was terminated and the remaining mice were euthanized with CO_2_ asphyxiation. For the ex vivo immunology experiments, mice were euthanized at the indicated time points.

### CD4^+^ T cell depletion

The anti-CD4 monoclonal antibody GK1.5 (Cell Culture Company, Minneapolis, MN) was used to deplete CD4^+^ T cells. Mice received the indicated dose of GK1.5 intraperitoneally in 100 μl PBS. Control mice received 100 μl intraperitoneal PBS. CD4^+^ T cell counts in peripheral blood were assessed by performing cheek bleeds in uninfected BALB/c mice. Four mice per dosage group were studied, so two mice per group were assessed each week and 2 weeks elapsed between blood collections from the same mice. For each sample, 100 μl whole blood was obtained and divided into technical duplicates for staining with CD3-PE (1:200 dilution, Cat #100308), CD4-PerCP-Cyanine5.5 (1:200 dilution, Cat #116012), and CD8-APC antibodies (1:200 dilution, Cat #100712) (BioLegend, San Diego, CA), followed by red blood cell lysis and fixation with 1 ml 1× RBC Lysis/Fixation Solution (BioLegend). Cells were then centrifuged at 350 x g for 5 min after which the pellets were resuspended with 275 μl FACS buffer and 25 μl of CountBright Absolute Counting Beads. Cells were analyzed on a 5-laser Bio-Rad ZE5 flow cytometer.

### Single-cell suspension preparation and lung CFU detection

Following anesthesia with isoflurane, blood was drawn by cardiac puncture with heparinized syringes and pooled within experimental groups. The blood was diluted with an equal volume of PBS containing 2% FBS, then layered over 5 ml of Ficoll-Paque PREMIUM 1.084 (Cytiva, Uppsala, Sweden) in 15 ml SepMate-50 tubes (STEM Cell Technology, Cambridge, MA). After centrifugation at 1200 × *g* for 20 min, the interphase containing PBMCs was collected. Spleens and lungs were dissociated after cardiac puncture and analyzed individually. Spleens were pressed with the piston of a 3 ml syringe and through a 70 µm cell strainer with 6 ml complete media to collect single cells. Lung single-cell suspensions were prepared using the MACS Lung Dissociation Kit for mouse as described by the manufacturer (Miltenyi Biotec, Bergisch Gladbach, Germany). Single-cell suspensions of the lungs were enriched for leukocytes using a 67 and 40% Percoll (Cytiva) gradient, followed by the collection of interphase cells. Single cells from the blood, spleen, and lungs were washed and resuspended in a complete culture medium supplemented with amphotericin B. The concentration and viability of cells were determined with Trypan blue (Bio-Rad) using a T20 auto cell counter. For lungs collected at 10 dpi or 70 dpi, undiluted and diluted lung single-cell suspensions were plated on Sabouraud dextrose agar, and incubated at 30 °C for 2–3 days, at which time CFUs of *Cryptococcus* were counted. The detection limit was 20 CFU/lung.

### Ex vivo cell culture and stimulation

PBMCs, splenocytes, and lung leukocytes were plated in tissue culture-treated 96-well round bottom plates at 2 × 10^5^, 1 × 10^6^, and 4 × 10^5^ cells/well, respectively. Cells were left unstimulated or stimulated with Cda1 or Cda2 protein (5 µg/ml), HK *C. neoformans* strain KN99 (50 µg/ml), or Staphylococcal Enterotoxin B (SEB, 1 µg/ml; Toxin Technology Inc, Sarasota, FL). Incubations were performed at 37 °C in humidified air supplemented with 5% CO_2_. PBMCs and splenocytes were cultured 3 days following which supernatants were collected and stored at −80 °C pending assay for IFNγ. For lung leukocytes, cells were cultured for 18 h. Brefeldin A (5 µg/ml, BioLegend) was added for the last 4 h of lung leukocytes and PBMCs culture. Duplicates or triplicates were conducted for each sample. Culture supernatants were collected and stored at −80 °C for subsequent IFNγ measurements, while the lung leukocytes were analyzed by flow cytometry.

### Quantification of IFNγ production

IFNγ concentrations in cell supernatants were determined with the R&D Systems Mouse IFNγ DuoSet ELISA Kit (Bio-Techne, Minneapolis, MN) according to the manufacturer’s protocol. Samples were run in 1:2 dilutions. The detection limit was 10 pg/ml. Values lower than the detection limit were arbitrarily assigned a value of 9 pg/ml.

### Intracellular staining and FACS analysis

Lung leukocytes were stained with a LIVE/DEAD green fixable dead cell stain kit (1:1000 dilution, Cat #L34970). Cell surface antigens were then stained with CD3-PE (1:200 dilution, Cat #100308), CD4-PerCP/Cyanine5.5 (1:200 dilution, Cat #100434), and CD8-APC (1:200 dilution, Cat #100712) (BioLegend). After fixation and permeabilization using the Intracellular Fixation & Permeabilization Buffer Set, cells were co-incubated with rat anti-mouse CD16/CD32 monoclonal antibody 2.4G2 (BD Pharmingen, 1:250 dilution, Cat #553141) to block Fc receptors and then stained with CD154-PE/Cyanine7 (1:400 dilution, Cat #106512), IFNγ-BV650 (1:200 dilution, Cat #505832), IL-17A-BV510 (1:200 dilution, Cat #506933), and TNFα-APC/Cyanine7 (1:400 dilution, Cat #506344) (BioLegend). FACS data were acquired with a 5-laser BD LSRII flow cytometer and analyzed using FlowJo version 10.8 software (BD, Franklin Lakes, NJ). Gating was established using FMO controls and isotype controls. Briefly, a singlet gate was created on a plot of forward scatter height (FSC-H) vs. forward scatter area (FSC-A). Then, debris were excluded based on FSC-A and side scatter area (SSC-A). The dead cells were excluded using the LIVE/DEAD green signal, the CD4^+^CD8^−^ population was selected from the CD3^+^ population, and finally, the expression of IFNγ, IL-17A, TNFα, and CD154 by the live CD3^+^CD4^+^CD8^−^ gated population was examined (see Supplementary Fig. 1). An identical gating strategy was used to examine CD8^+^ T cells except the CD4^-^CD8^+^ population was selected from the CD3^+^ population.

### Statistics

Data were analyzed and graphs were drawn using GraphPad Prism, version 9.2.0 (GraphPad Software, La Jolla, CA). Kaplan–Meier survival curves were analyzed for significance using the Mantel–Cox log-rank test. When median survival differences were ≤3d, findings were not considered biologically significant and thus not denoted as statistically significant. Lung CFUs were compared using the Mann–Whitney test. Lung leukocytes, CD4^+^ T cell, and CD8^+^ T cell numbers in groups were compared using the one-way ANOVA test with Bonferroni’s correction for multiple comparison. For the ex vivo experiments, technical triplicates of pooled PBMC in each group were presented and analyzed. Averages of technical duplicates or triplicates for each sample were presented and data were analyzed individually for the spleen and lungs. The comparisons of cytokine expression among groups were performed by two-way ANOVA test with Bonferroni’s correction applied for multiple comparisons. Significance was defined as a *P* value of < 0.05 following corrections for multiple comparisons.

## Supplementary information

Supplementary Material

## Data Availability

The datasets generated during and/or analyzed during the current study are available from the corresponding author on reasonable request.
