# Supplementary material for: Immunological correlates of protection following vaccination with glucan particles containing Cryptococcus neoformans chitin deacetylases
Source: NPJ Vaccines. 2023 Feb 2;8:6. doi: 10.1038/s41541-023-00606-0 (PMC9892683; doi:10.1038/s41541-023-00606-0)
Supplement: Supplementary file 1 — Supplementary Material [file 41541_2023_606_MOESM1_ESM.pdf]

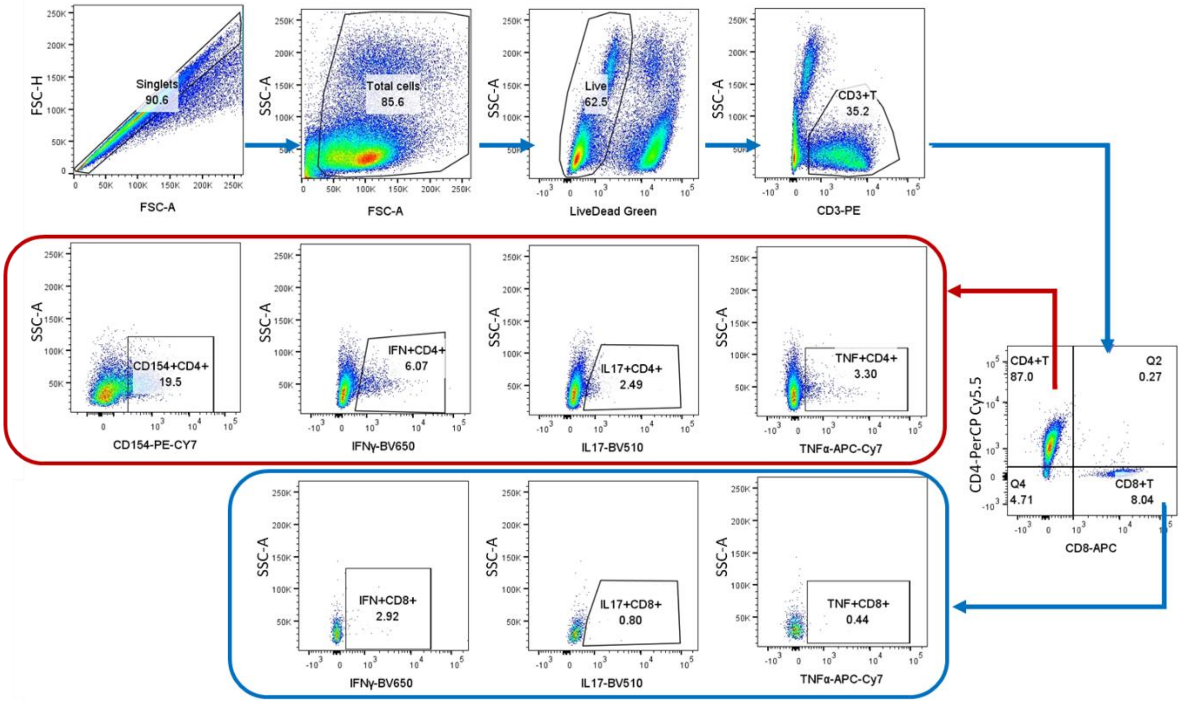

Supplementary Figure 1. Representative flow cytometry plots illustrating the gating strategy for experiments examining T cell activation and intracellular cytokine production in cultured lung leukocytes. Singlet cells were gated based on forward scatter (FSC) height (FSC-H) vs. area (FSC-A). Debris was excluded based on FSC-A and side scatter area (SSC-A). Dead cells were excluded based on LIVE/DEAD green staining. T cells were selected based on $CD3^+$ staining. The $CD4^+CD8^-$ population was selected from the $CD3^+$ population. Finally, the intracellular expression of IFNγ, IL-17A, TNFα and CD154 by the live $CD3^+CD4^+CD8^-$ gated population (shown in red box) was examined. An identical gating strategy was used to examine $CD8^+$ T cells except the $CD4^-CD8^+$ population (shown in blue box) was selected from the $CD3^+$ population. CD154 expression was not analyzed in the $CD4^-CD8^+$ population. The plots are from lung cells of a GP-Cda1/Cda2 vaccinated mouse 10d post infection stimulated *ex vivo* with SEB.

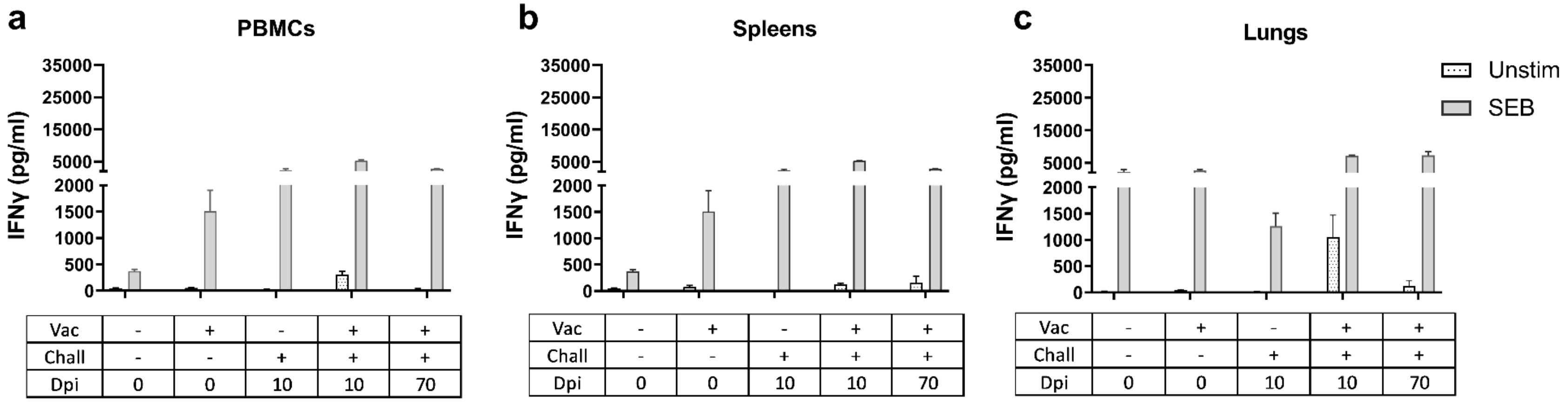

Supplementary Figure 2. IFNγ production by PBMCs, splenocytes, and lung leukocytes following SEB stimulation. Experiments were designed and conducted as indicated in the Fig. 5 legend. Supernatants of unstimulated (Unstim) or SEB-stimulated PBMCs (**a**), spleens (**b**) and lung (**c**) were collected and analyzed for IFNγ by ELISA. Each group had 5 mice. Vac, vaccinated with GP-Cda1/Cda2. Chall, challenged with *C. neoformans* strain KN99. Dpi, days post infection. The results of the statistical comparisons between unstimulated and SEB-stimulated groups are shown in Supplementary Figure 3.

a. PBMCs

Group	Unstim vs Cda1		Unstim vs Cda2		Unstim vs KN99		Unstim vs SEB	
**Vac- / Chall- / Dpi 0**	ns	>0.9999	ns	>0.9999	ns	>0.9999	ns	>0.9999
**Vac+ / Chall- / Dpi 0**	*	0.0268	****	<0.0001	ns	>0.9999	****	<0.0001
**Vac- / Chall+ / Dpi 10**	ns	>0.9999	**	0.0061	ns	>0.9999	****	<0.0001
**Vac+ / Chall+ / Dpi 10**	****	<0.0001	****	<0.0001	**	0.0019	****	<0.0001
**Vac+ / Chall+ / Dpi 70**	ns	0.7014	ns	0.0769	***	0.0003	****	<0.0001

b. Spleens

Group	Unstim vs Cda1		Unstim vs Cda2		Unstim vs KN99		Unstim vs SEB	
**Vac- / Chall- / Dpi 0**	ns	>0.9999	ns	>0.9999	ns	>0.9999	****	<0.0001
**Vac+ / Chall- / Dpi 0**	***	0.0005	****	<0.0001	**	0.0011	****	<0.0001
**Vac- / Chall+ / Dpi 10**	ns	>0.9999	ns	>0.9999	ns	>0.9999	ns	0.1228
**Vac+ / Chall+ / Dpi 10**	****	<0.0001	**	0.0034	ns	>0.9999	****	<0.0001
**Vac+ / Chall+ / Dpi 70**	****	<0.0001	****	<0.0001	****	<0.0001	****	<0.0001

c. Lungs

Group	Unstim vs Cda1		Unstim vs Cda2		Unstim vs KN99		Unstim vs SEB	
**Vac- / Chall- / Dpi 0**	ns	>0.9999	ns	>0.9999	ns	>0.9999	ns	0.0958
**Vac+ / Chall- / Dpi 0**	*	0.0186	***	0.0001	ns	>0.9999	**	0.0052
**Vac- / Chall+ / Dpi 10**	ns	>0.9999	ns	>0.9999	ns	>0.9999	ns	0.4789
**Vac+ / Chall+ / Dpi 10**	****	<0.0001	****	<0.0001	****	<0.0001	****	<0.0001
**Vac+ / Chall+ / Dpi 70**	***	0.0001	****	<0.0001	***	0.0004	****	<0.0001

Supplementary Figure 3. Statistical comparisons between groups in Fig. 5 and Supplementary Figure 2. Experiments were designed and conducted as indicated in the Fig. 5 and Supplementary Figure 2 legends. IFNγ production of unstimulated cells and cells stimulated with the indicated antigens was compared using two-way ANOVA with Bonferroni's correction. Statistics of comparison were shown in **a** for PBMCs, **b** for Spleens and **c** for Lungs. Vac, vaccinated with GP-Cda1/Cda2. Chall, challenged with *C. neoformans* strain KN99. Dpi, days post infection. Unstim, unstimulated. ns (not significant), $P > 0.05$. *, $P < 0.05$. **, $P < 0.005$. ***, $P < 0.0005$. ****, $P < 0.0001$. Comparisons with statistical differences are shown in red font.

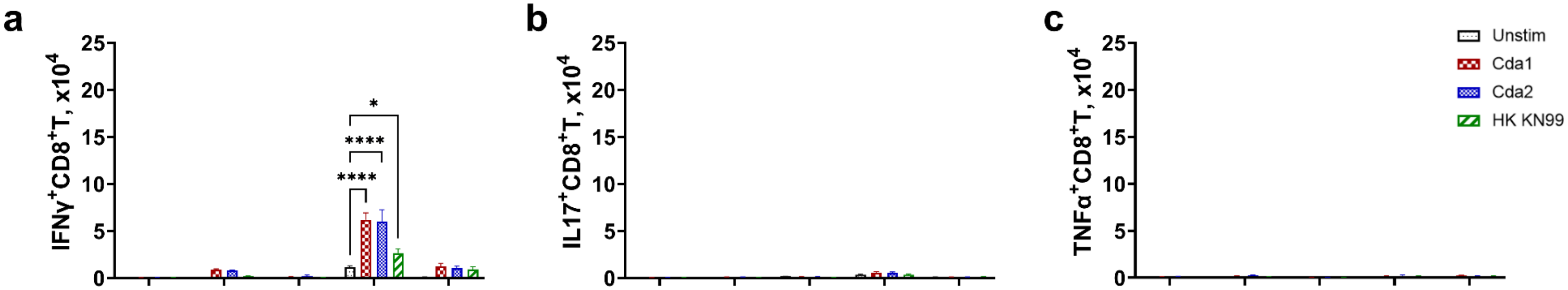

Vac	-	+	-	+	+
Chall	-	-	+	+	+
Dpi	0	0	10	10	70

Vac	-	+	-	+	+
Chall	-	-	+	+	+
Dpi	0	0	10	10	70

Vac	-	+	-	+	+
Chall	-	-	+	+	+
Dpi	0	0	10	10	70

Supplementary Figure 4. Intracellular cytokine production by pulmonary CD8$^+$ T cells in GP-Cda1/Cda2-vaccinated and/or infected mice. BALB/c mice were vaccinated thrice at biweekly intervals with GP-Cda1/Cda2. Two weeks after last boost, the mice received a pulmonary challenge with *C. neoformans*. Mice were euthanized at 0 dpi (uninfected), 10 dpi or 70 dpi. Controls included unvaccinated mice euthanized at 0 dpi or 10 dpi. Lungs were harvested and single cell suspensions were prepared. Leukocytes were cultured in complete media supplemented with amphotericin B and stimulated with the indicated antigens or left unstimulated (Unstim) for 18h. Then the cells were collected, stained, and analyzed by polychromatic FACS, as described in Methods. From left to right shows the numbers of lung CD8$^+$ T cells producing the intracellular cytokines IFNγ (**a**), IL-17 (**b**), and TNFα (**c**) following *ex vivo* stimulation. Each group had 5 mice. Vac = vaccinated with GP-Cda1/Cda2, Chall = challenged with *C. neoformans*, Dpi = days post infection, HK = heat killed. Two-way ANOVA with Bonferroni's correction was used for comparisons. *, $P < 0.05$. ****, $P < 0.0001$.

a. CFUs

Group	Summary	*P* Value
**Vac- / Chall+ / Dpi 10 vs Vac+ / Chall+ / Dpi 10**	ns	0.0952
**Vac- / Chall+ / Dpi 10 vs Vac+ / Chall+ / Dpi 70**	**	0.0079
**Vac+ / Chall+ / Dpi 10 vs Vac+ / Chall+ / Dpi 70**	**	0.0079

b. Leukocytes/CD4$^+$ T/ CD8$^+$ T cell counts

Group	Leukocytes		CD4$^+$T		CD8$^+$T	
**Vac- / Chall- / Dpi 0 vs Vac- / Chall+ / Dpi 10**	ns	0.4785	ns	0.7759	ns	>0.9999
**Vac- / Chall- / Dpi 0 vs Vac+ / Chall- / Dpi 0**	ns	>0.9999	ns	>0.9999	ns	>0.9999
**Vac- / Chall- / Dpi 0 vs Vac+ / Chall+ / Dpi 70**	ns	>0.9999	ns	>0.9999	ns	>0.9999
**Vac- / Chall- / Dpi 0 vs Vac+ / Chall+ / Dpi 10**	****	<0.0001	****	<0.0001	***	0.0002
**Vac- / Chall+ / Dpi 10 vs Vac+ / Chall+ / Dpi 10**	****	<0.0001	****	<0.0001	**	0.0016
**Vac+ / Chall- / Dpi 0 vs Vac+ / Chall+ / Dpi 10**	****	<0.0001	****	<0.0001	**	0.0011
**Vac+ / Chall+ / Dpi 10 vs Vac+ / Chall+ / Dpi 70**	****	<0.0001	****	<0.0001	***	0.0004
**Vac+ / Chall- / Dpi 0 vs Vac+ / Chall+ / Dpi 70**	ns	>0.9999	ns	>0.9999	ns	>0.9999

Supplementary Figure 5. Statistical comparison for lung CFU and cell numbers in Fig. 6. Experiments were designed and conducted as indicated in Fig. 6 legend. Statistics of comparison were shown in (**a**) for lung CFUs (Man-Whitney test); (**b**) for lung leukocyte / CD4+T / CD8+T numbers (One-way ANOVA with Bonferroni's correction); (**c**) for CD154$^+$CD4$^+$T numbers, (**d**) for IFNγ$^+$CD4$^+$T numbers; (**e**) for IL-17$^+$CD4$^+$T numbers; and (**f**) for TNFα$^+$CD4$^+$T numbers (Two-way ANOVA with Bonferroni's correction were used for **c-f**). Vac, vaccinated with GP-Cda1/Cda2. Chall, challenged with *C. neoformans* strain KN99. Dpi, days post infection. Unstim, unstimulated. ns (not significant), $P > 0.05$. *, $P < 0.05$. **, $P < 0.005$. ***, $P < 0.0005$. ****, $P < 0.0001$. Comparisons with statistical differences are shown in red font.

c. CD154$^+$CD4$^+$ T

Group	Unstim vs Cda1		Unstim vs Cda2		Unstim vs KN99	
**Vac- / Chall- / Dpi 0**	ns	>0.9999	ns	>0.9999	ns	>0.9999
**Vac+ / Chall- / Dpi 0**	ns	>0.9999	ns	>0.9999	ns	>0.9999
**Vac- / Chall+ / Dpi 10**	ns	>0.9999	ns	>0.9999	ns	>0.9999
**Vac+ / Chall+ / Dpi 10**	****	<0.0001	****	<0.0001	****	<0.0001
**Vac+ / Chall+ / Dpi 70**	ns	>0.9999	ns	>0.9999	ns	>0.9999

d. IFNγ$^+$CD4$^+$ T

Group	Unstim vs Cda1		Unstim vs Cda2		Unstim vs KN99	
**Vac- / Chall- / Dpi 0**	ns	>0.9999	ns	>0.9999	ns	>0.9999
**Vac+ / Chall- / Dpi 0**	ns	>0.9999	ns	>0.9999	ns	>0.9999
**Vac- / Chall+ / Dpi 10**	ns	>0.9999	ns	>0.9999	ns	>0.9999
**Vac+ / Chall+ / Dpi 10**	****	<0.0001	****	<0.0001	****	<0.0001
**Vac+ / Chall+ / Dpi 70**	ns	>0.9999	ns	>0.9999	ns	>0.9999

e. IL-17$^+$CD4$^+$ T

Group	Unstim vs Cda1		Unstim vs Cda2		Unstim vs KN99	
**Vac- / Chall- / Dpi 0**	ns	>0.9999	ns	>0.9999	ns	>0.9999
**Vac+ / Chall- / Dpi 0**	ns	>0.9999	ns	>0.9999	ns	>0.9999
**Vac- / Chall+ / Dpi 10**	ns	>0.9999	ns	>0.9999	ns	>0.9999
**Vac+ / Chall+ / Dpi 10**	****	<0.0001	****	<0.0001	****	<0.0001
**Vac+ / Chall+ / Dpi 70**	ns	>0.9999	ns	>0.9999	ns	>0.9999

f. TNFα$^+$CD4$^+$ T

Group	Unstim vs Cda1		Unstim vs Cda2		Unstim vs KN99	
**Vac- / Chall- / Dpi 0**	ns	>0.9999	ns	>0.9999	ns	>0.9999
**Vac+ / Chall- / Dpi 0**	ns	0.1329	ns	>0.9999	ns	>0.9999
**Vac- / Chall+ / Dpi 10**	ns	>0.9999	ns	>0.9999	ns	>0.9999
**Vac+ / Chall+ / Dpi 10**	****	<0.0001	****	<0.0001	****	<0.0001
**Vac+ / Chall+ / Dpi 70**	ns	>0.9999	ns	>0.9999	ns	>0.9999
